# Feasibility of Right Upper Transversal Hepatectomy in the Absence of an Inferior Right Hepatic Vein: New Insights regarding This Complex Procedure

**DOI:** 10.1155/2021/6668269

**Published:** 2021-03-06

**Authors:** Fabio Ferrari Makdissi, Jaime Arthur Pirola Kruger, Vagner Birk Jeismann, Paulo Herman

**Affiliations:** Digestive Surgery Division, Department of Gastroenterology, Instituto do Câncer do Estado de São Paulo (ICESP), University of São Paulo School of Medicine, Avenida Piassanguaba, 350, 04060-000 São Paulo, Brazil

## Abstract

**Background:**

Right upper transversal hepatectomy (RUTH) is defined as the removal of liver segments 7, 8, and 4A with ligature of the right and middle hepatic veins and is considered one of the most complex techniques of parenchymal-sparing hepatectomies. This procedure can be performed, without venous reconstruction, if collateral veins are present communicating within remnant liver segments to a large inferior right hepatic vein and/or to the left hepatic vein. This venous network could maintain outflow from the inferior right segments (S5, S6) to the left liver when a RUTH is performed, even in the absence of an inferior right hepatic vein. The aim of this study is to present our experience with RUTH without venous reconstruction in patients with and without the presence of an inferior right hepatic vein (IRHV).

**Methods:**

Patients submitted to RUTH for treatment of liver metastases were selected from our database. The presence of an IRHV, clinical and surgical characteristics of the patients, immediate outcomes, viability of liver segments 5 and 6, and long-term survival were analyzed.

**Results:**

RUTH was successfully performed in four patients. In two patients, IRHV was not present, but intrahepatic communicating veins between proximal right and middle hepatic veins and left hepatic vein were present. No venous reconstructions were performed. Mild congestion of the inferior right segments occurred in the patients where there was no IRHV but no immediate, early, or late complications were observed.

**Conclusions:**

RUTH is feasible and can be performed even in the absence of an IRHV, without venous reconstruction. Some degree of congestion of the right inferior liver segments might occur when an IRHV is absent, yet this is not clinically significant when communicating veins are present. Maximum parenchyma preservation might prevent postoperative liver failure and allow repeated resections in case of hepatic recurrence.

## 1. Introduction

**Figure 1:**
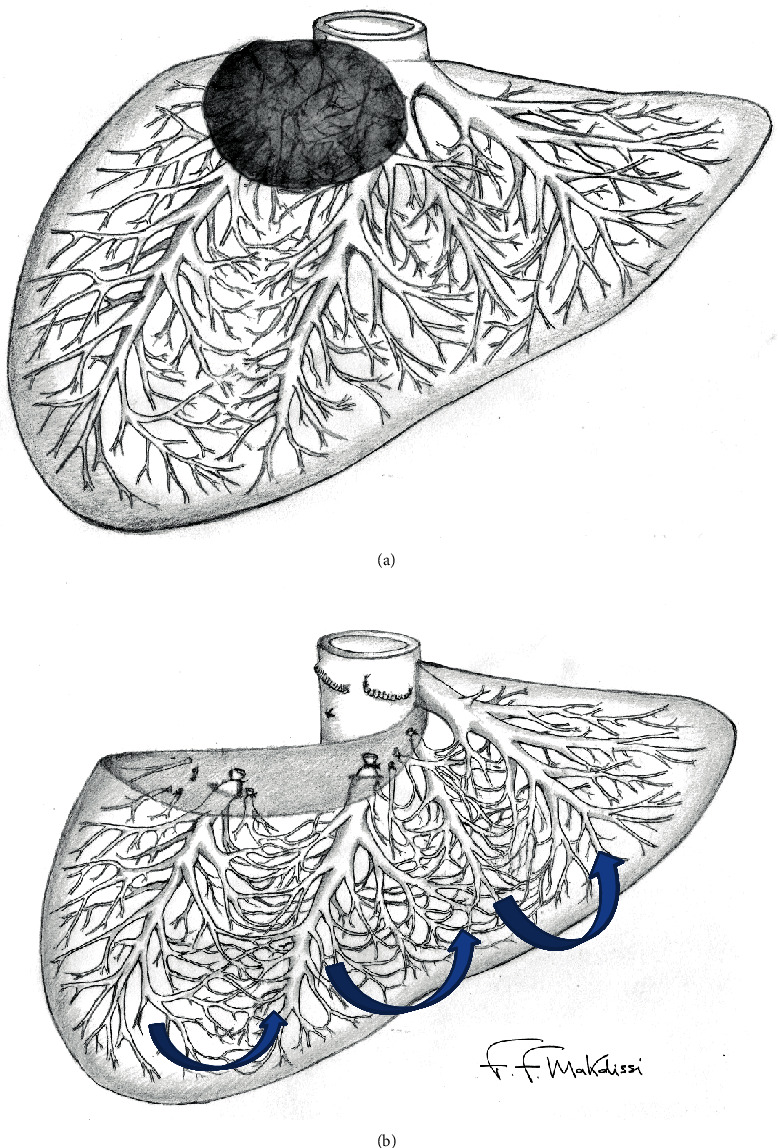
Collateral vein network in the absence of IRHV: (a) tumor located in segments 7-8 and 4A, with close relation or involvement of the right hepatic vein and middle hepatic vein. (b) Venous outflow through collaterals from the right to left liver.

Parenchymal-sparing hepatectomies are increasingly used as alternatives to major liver resections, mainly for treatment of liver metastases. The purpose of this approach is to achieve oncologic resection while preserving as much parenchyma as possible [1–10]. Parenchymal-sparing hepatectomies may decrease the risk of post-operative liver failure with better short-term outcomes than major liver resections, and similar oncologic results [2, 5, 6]. Another advantage is to favor rehepatectomies with curative intent for intrahepatic recurrence of colorectal metastases, associated with favorable long-term outcomes [11–13]. Among those parenchymal-sparing techniques, the extended right superior liver resection, which is also known as “right upper transversal hepatectomy,” is one of the most complex described [8, 9]. Right upper transversal hepatectomy (RUTH) is defined as the removal of liver segments 7, 8, and 4A with ligature of the right and middle hepatic veins [8–10]. This procedure is feasible if intrahepatic collateral veins are present communicating proximal right and middle hepatic veins with an inferior right hepatic vein and the left hepatic vein; however, it can be performed, even in the absence of a large inferior right hepatic vein (IRHV), in the presence of communicating veins to the left hepatic vein (Figures 1(a) and 1(b)) [9, 10]. The presence of communicating veins is necessary to avoid congestion of the remnant inferior right segments due to impairment of the venous outflow.

Feasibility of RUTH without hepatic vein reconstruction, in the presence or absence of the right inferior hepatic vein, has already been mentioned; however, the number of cases described in the literature is scarce [9, 10].

The aim of this study is to present our experience with RUTH without any type of venous reconstruction in patients with and without the presence of an IRHV.

## 2. Methods

We reviewed retrospectively all patients enrolled in our prospective database, submitted to liver resection and underwent RUTH from 2009 to 2020, at the Cancer Institute of the University of São Paulo (Department of Gastroenterology).

Clinical and surgical characteristics of the patients, immediate and early outcomes, viability of liver segments 5 and 6, and long-term survival were analyzed.

### 2.1. Preoperative Assessment

Patients were submitted to preoperative 3 phase cross-sectional computed tomography or magnetic resonance imaging. Relationship between tumor and right and middle hepatic veins was evaluated, as well as the caliber and patency of these veins. Volumetry of left lateral segments and caudate lobe were calculated and should have at least 25% of total liver volume, in case of conversion to a major resection.

Patients should have a preserved liver function on clinical and laboratory tests (Child-Pugh score 5).

All cases were previously discussed in multidisciplinary meetings which included liver surgeons, radiologists, and clinical oncologists. Whenever possible, our group's policy favors liver-sparing resections while always respecting oncological principles. The RUTH procedure was proposed when the tumor was closely related to or involving the right and middle hepatic veins, near the hepatocaval confluence, and in the presence of an IRHV (more than 0.5 cm in diameter). In the absence of an IRHV, RUTH procedure was proposed when the lumen of the right and middle hepatic veins near the hepatocaval confluence was more than 2/3 of its compromised size, understanding that a compensatory collateral network of venous drainage had already been developed.

### 2.2. Surgical Technique

A right subcostal incision that extended superiorly to the midline of the xyphoid was performed. After laparotomy, a self-retaining retractor was used. The liver was mobilized by sectioning round, falciform, right triangular, and coronary ligaments. The right side of the retrohepatic cava vein was exposed, with division of the cava ligament but without ligature of the right inferior branches. When an inferior right hepatic vein is present, caution should be taken not to damage it. An intraoperative ultrasound was performed to identify the liver metastasis and to plan the liver resection line with an appropriate oncologic margin. At this time, the course of the right and middle hepatic vein can be checked as well as the major glissonian pedicles inside the liver. Larger vascular structures are identified and demarcated on the surface of the liver with an electric scalpel. The distal right and middle hepatic veins were isolated outside the liver at the cava confluence. For access and isolation of the middle hepatic vein, which usually joins the left hepatic vein within the liver in a single trunk, a small opening of the hepatic parenchyma is performed between these two veins until the confluence is found. Distal right and middle hepatic veins were clamped before liver transection for a “clamping test,” and preservation of portal inflow and venous outflow was confirmed through color-Doppler ultrasonography in the future remnant segments. Clamping of hepatic veins was released for liver transection. The parenchyma was transected under an intermittent Pringle maneuver within the predefined line, and oncological margin was frequently checked by ultrasound. For liver transection, CUSA (Cavitron Ultrasonic Surgical Aspirator; ValleyLab, Boulder, Colorado, USA) and/or bipolar energy associated with ligature and section of major portal pedicles and hepatic veins are performed. The latter step is the division of the right and middle hepatic veins at the caval confluence between vascular clamps or with an endoscopic vascular linear stapler. After the resection is complete, color-Doppler ultrasonography is performed to confirm vascular perfusion and drainage of remaining liver segments. A suction silicone drain (size 19 French) was placed.

### 2.3. Follow-Up

The follow-up of patients submitted to surgical treatment of liver metastases in our group includes computed tomography or magnetic resonance with intravascular contrast every 4 months for the first 2 years after hepatectomy, and every 6 months thereafter.

## 3. Results

From 2009 to 2020, there were 628 liver resections at the Cancer Institute of the University of São Paulo, of which 491 were hepatectomies for liver metastases treatment. Four patients (3 females and 1 male, with a mean age of 62.7 years) were operated for colorectal liver metastasis and underwent RUTH without any type of venous reconstruction. Two patients presented a large IRHV (Patients 1 and 2), and two presented no significant IRHV (Patients 3 and 4).

The patients' data are summarized in Table 1.

### 3.1. Patient 1

**Figure 2:**
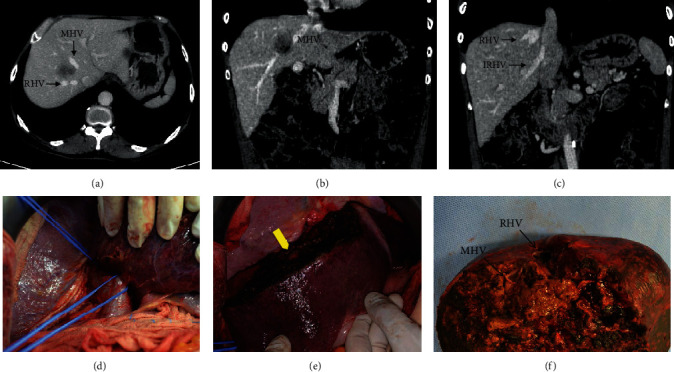
(Patient 1) (a) Colorectal metastasis with no margin from RHV and MHV. (b) Local invasion of MHV. (c) Inferior right hepatic vein draining the right inferior segments (S5 and S6). (d) Liver mobilization and preservation of IRHV (asterisk). (e) Middle hepatic vein ligated (yellow arrow). (f) Surgical specimen (liver segments 4A, 8, and 7).

The preoperative CT scan disclosed a segment 8 tumor (3.5 cm) in close relationship with the right and middle hepatic veins, but these veins were still patent and normal in size, suggesting preserved vascular flow. As a large IRHV (1.2 cm diameter) was present to drain inferior right segments, a RUTH procedure was proposed instead of an R1 vascular resection to decrease the risk of compromised oncological margin. Another two lesions were present in segment 1 (1.3 cm) and segment 3 (0.9 cm), treated with wedge resections. No signs of liver congestion were seen during the liver resection (Figure 2). Estimated blood loss was 600 ml. Operative time was 330 minutes. Postoperative was uneventful, without clinical or laboratory liver failure. Patient was discharged on the 8th postoperative day. A late CT scan showed no signs of liver congestion, atrophy, or necrosis of the inferior liver segments 5 and 6 (Figure 2).

### 3.2. Patient 2

**Figure 3:**
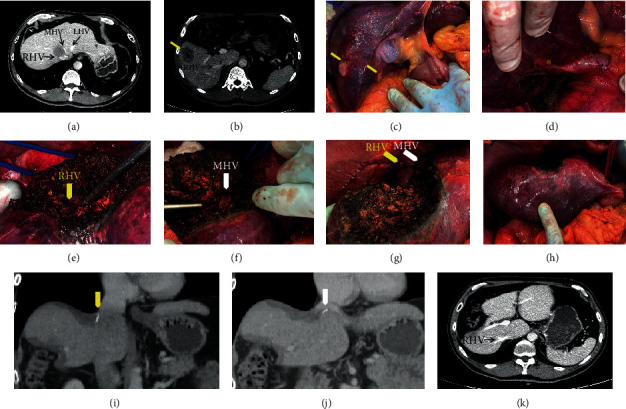
(Patient2) (a) Colorectal metastasis with right and middle hepatic vein invasion. (b) Inferior right hepatic vein draining the right inferior segments (S5 and S6). (c) Segment 5 liver metastasis and post FOLFOX “blue liver aspect.” (d) Liver mobilization and preservation of IRHV (asterisk). (e) Right hepatic vein isolation during liver transection (yellow arrow). (f) Middle hepatic vein isolation during liver transection (white arrow). (g) Immediate postoperative aspects of the liver, distal right (yellow arrow), and middle hepatic vein (white arrow) ligated at the vena cava confluence. (h) Right upper transversal hepatectomy and segment 5 resection. (i, j) Postoperative CT: right (yellow arrow) and middle hepatic vein (white arrow). (k) Postoperative CT: inferior right hepatic vein draining segment 6.

Preoperative CT scan disclosed a segment 7/8 tumor (3.9 cm), and the right and middle hepatic veins had a significant reduction in size (greater than 2/3 of their caliber) next to the cava confluence, suggesting compromised flow. This patient had a large IRHV (0.9 cm diameter) draining inferior right segments, and a RUTH procedure was performed. Another two lesions were present in segment 5 (4.1 and 2 cm) and were resected with a segment 5 wedge resection. No signs of liver congestion were seen during the liver resection (Figure 3). Estimated blood loss was 100 ml. Operative time was 420 minutes. Postoperative was uneventful, without clinical or laboratory liver failure. Postoperative hospital stay was of 6 days. No signs of liver congestion, atrophy, or necrosis in the inferior liver segments 5 and 6 were present in the postoperative CT scan (Figure 3).

### 3.3. Patient 3

**Figure 4:**
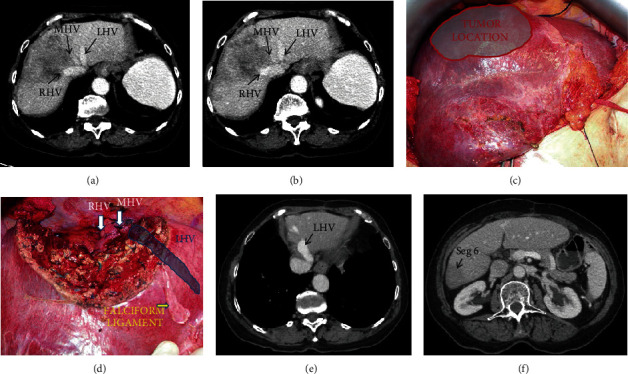
(Patient 3) (a, b) Colorectal liver metastasis with right and middle hepatic vein invasion. (c) Intraoperative aspect of the liver and tumor location. (d) Immediate postoperative aspects of the liver, distal right, and middle hepatic vein ligated at the vena cava confluence. (e) Left hepatic vein draining remnant liver segments. (f) Postoperative segment 6 atrophy.

Preoperative CT scan disclosed a segment 7/8/4A tumor (7.2 cm); the right hepatic and middle hepatic veins were completely involved by the tumor with significant reduction of their size at the cava confluence (greater than 2/3 of their caliber), suggesting compromised flow. RUTH procedure was proposed, considering compensatory outflow through intrahepatic communicating veins of inferior segments. Intraoperatively, a preresection clamping test of the right and middle hepatic veins with color-Doppler ultrasonography showed preserved portal inflow to the right inferior segments and venous drainage throughout collateral veins. A mild congestion of segment 6 was observed immediately after resection, but there was no increased bleeding from the raw surface. Post resection, intraoperative color-Doppler ultrasound showed maintenance of vascular inflow and outflow of the right inferior segments. Estimated blood loss was 400 ml and operative time was 360 minutes. Postoperative was uneventful, with no blood transfusion or liver function abnormality. A postoperative CT scan disclosed a slight congestion of segment 6, but the liver parenchyma volume of other remnant segments was preserved, with blood outflow through the left hepatic vein (Figure 4). This patient had a liver recurrence 16 months later and was successfully submitted to a segment 3 wedge resection.

### 3.4. Patient 4

**Figure 5:**
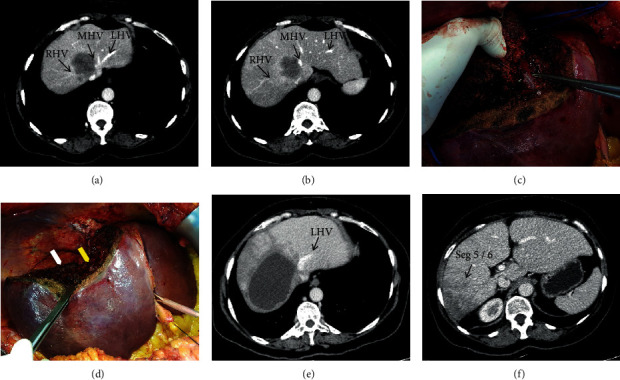
(Patient 4) (a, b) Colorectal liver metastasis with right and middle hepatic veins invasion. (c) Middle hepatic vein isolation (asterisk) during liver transection. (d) Immediate postoperative aspects of the liver with proximal right and middle hepatic vein ligature (white and yellow arrows, respectively). (e) Left hepatic vein draining remnant liver segments. (f) Postoperative segments 5 and 6 atrophy.

A segment 7/8/4A tumor (6.0 cm) was seen on the preoperative CT scan; the right hepatic vein was completely involved by the tumor, and the main trunk of the middle hepatic vein had a significant size reduction at the cava confluence, suggesting compromised flow. RUTH procedure was proposed, considering compensatory outflow through intrahepatic communicating veins. The intraoperative preresection clamping test of right and middle hepatic veins revealed preserved portal inflow to inferior right liver segments and outflow through collateral veins. After hepatectomy, an intraoperative mild congestion of both of the inferior right segments (S5 and S6) was present, but no uncontrollable bleeding occurred in the raw surface (Figure 5). The intraoperative color-Doppler ultrasound showed maintenance of vascular inflow, and the venous outflow was present in larger veins. No hemodynamic instability occurred. Estimated blood loss was 200 ml and operative time was 300 minutes. Postoperative was uneventful, with no blood transfusion or liver dysfunction, and the patient was discharged on the 7th postoperative. A late postoperative CT scan disclosed the venous outflow of the remnant liver through the left hepatic vein, minor congestion and atrophy of liver segments 5 and 6, and hypertrophy of the left liver (Figure 5).

## 4. Discussion

The parenchymal-sparing technique is considered an oncologic resection and is currently recommended as an alternative to major hepatectomies. This approach aims a maximum parenchymal preservation, decreasing the risk of postoperative liver failure enabling rehepatectomies in case of liver recurrence [1–8]. Tumor characteristics such as number, size, and proximity to large vessels are the main factors that determine the feasibility of this approach. One of the most challenging surgical conditions is when tumors are located in segments 7-8 and 4A, either with close relation to or involvement of the right hepatic vein and middle hepatic vein (Figure 1(a)). In those cases, an extended right hepatectomy after preoperative right portal vein embolization may be required [14]. The options to spare liver parenchyma are (1) tumor enucleation with vascular (right and middle hepatic veins) detachment, (2) right upper transversal hepatectomy with hepatic vein reconstruction, or (3) right upper transversal hepatectomy without venous reconstruction.

Liver resections with detachment from major hepatic veins, the so-called R1 vascular resection, present coincident margins to major intrahepatic vessels but may have similar results to free margins (R0 resections) [15]. These authors suggest that tumor can be resected preserving major hepatic veins whenever the contact is up to two-thirds and no clear invasion is present. They also propose that maximal liver preservation with expanded and complex techniques, frequently including R1 vascular resections, may yield to satisfactory oncological results [15, 16]. This technique must be avoided for lesions involving more than two-thirds of the vessel's circumference, due to a high probability of tumor exposure or vascular damage. Therefore, for lesions on segments 7-8-4A with invasion of the right and middle hepatic veins or more than two-thirds of circumferential involvement, liver resections with or without hepatic vein reconstruction are the options when intending to preserve the inferior right segments.

In 2016, Alvarez et al. published a very interesting compilation of parenchymal-sparing liver resection strategies and mentioned the “en bloc” resection of segments 7, 8, and 4A (with or without segment 2) and resection of right and middle hepatic veins as one of the most complex techniques [8]. Torzilli et al. first proposed this “Upper Transversal Hepatectomy” approach [10] and lately subdivided it in right, left, and total upper transversal hepatectomy [9]. This liver-sparing technique is rarely employed, as the remaining inferior segments of the liver would present outflow impairment, leading to liver congestion and failure.

Back in the day, Makuuchi et al. described a large inferior right hepatic vein, an accessory hepatic vein of the right liver (posteriorly named after him), that can drain segments 5 and 6 when a right superior liver resection (segments 7 and 8) is performed with right hepatic vein ligation [17]. The success of this technique in early reports was obtained if a Makuuchi's vein was present. The right superior liver resection or bisegmentectomy 7-8 along with resection of the right hepatic vein was proved to be feasible [18–20]. Later on, some authors showed its feasibility, even in the absence of an IRHV since outflow may be directed through collateral veins into the middle hepatic vein, thus maintaining the venous drainage of segments 5 and 6 avoiding congestion [21, 22]. The nomenclature “Mini-Upper Transversal Hepatectomy” was recently adopted to this technique [9]. Consequently, segment 6 can maintain its drainage through segment 5, and segment 5 can guarantee its drainage to the middle hepatic vein. The presence of communicating veins between adjacent hepatic segments was confirmed in several anatomical and radiological studies, mainly between the right and middle hepatic vein [23–28]. Despite having proven the existence of these intrahepatic communicating veins, the clinical impact for liver resections remains a discussion topic. Recently, Torzilli et al. have verified a map of the compensatory intrahepatic venous network between the IRHV, proximal right, proximal middle, and left hepatic veins using intraoperative ultrasonographic Doppler [10, 29]. They suggested that this collateral venous network may be broader and sufficient for satisfactory drainage of the remaining segments of the right liver when an upper transversal hepatectomy is performed, eliminating the need for hepatic vein reconstruction. This vein network could counterbalance the lack of drainage of the right and middle hepatic veins to the vena cava. As an alternative to major resections for colorectal liver metastases involving the hepatocaval confluence, Urbani et al. advocate the parenchymal-sparing hepatectomy including resection of major veins with vein reconstruction using a direct suture method or by interposition of a ringed polytetrafluoroethylene graft. An exception could be made for the need of RHV reconstruction when an IRHV is present. These authors referred to this technique as “Minor-but-complex.” This concept could have major significance when an “en block” resection of segments 7-8-4A is performed. Although it is considered a technique that allows maximum maintenance of well-perfused hepatic parenchyma, parenchymal-sparing resections with vascular reconstruction can be very complex and must be performed by experienced groups. These authors justify the importance of the reconstruction of hepatic veins in an immediate to early period after resection to avoid complications such as liver congestion, bleeding, hemodynamic instability, and functional impairment of the spared liver. In the early to late postoperative period, this venous reconstruction may lose importance, since the rate of thrombosis of this graft is high yet does not exhibit clinical significance. They believe that vascular reconstruction with grafts increases safety, acting as a “bridge” until the maturation of venous collateral circulation between the hepatic segments [30, 31].

As the growth of neoplastic lesions is neither acute nor sudden, we hypothesized that vascular impairments inside the liver occur slowly and progressively, giving time to vascular compensation conditions of the surrounding parenchyma. The continuous and progressive involvement of the right and middle hepatic veins, related to tumors of the upper right segments, may lead to the development of a compensatory venous network, creating compensatory conditions for adequate drainage of the inferior segments that are not affected by the tumor. In fact, if the tumor is truly involving the hepatic veins and impairing its flow, there are only two possibilities: atrophy of the liver segments that depends on the drainage through these veins or compensation through a network of venous collaterals. If atrophy of the right inferior segments has occurred due to blockage of venous drainage, a major hepatic resection can be performed with low risk of liver failure, since the left segments must have had compensatory hypertrophy. In the presence of lesions with right and middle hepatic vein circumferential involvement, and significant reduction of their lumen (>2/3) near the cava confluence, a compensatory vascular network has probably already occurred if the inferior right segments are not atrophic. An increased risk of venous congestion of the remnant liver might occur if the tumor involves the right and middle hepatic veins but without venous flow impairment on these vessels. This can be preoperatively verified with radiologic exams, such as computed tomography or magnetic resonance with venous contrast, showing the hepatic veins with regular caliber and flow, absence of perfusion disorders, and nonappearance of inferior liver segment atrophy or perfusion disturbances. In this circumstance when an IRVH is absent, the RUTH procedure without venous reconstruction should be contraindicated.

In this report, we present four patients submitted to right upper transversal hepatectomy. Two patients had liver metastasis in contact with the right and middle hepatic veins close to the caval confluence, and a dominant inferior right hepatic vein was identified in the preoperative image exams. Two patients presented either circumferential involvement or tumor invasion of the right and middle hepatic veins, with significant caliber reduction, and no IRHV. None of these patients had preoperative radiologic evidence of atrophy of the right inferior segments, suggesting outflow through communicating veins within these segments. The RUTH procedures occurred without complications or blood transfusions. No signs of macroscopic congestion were observed in the patient with the accessory hepatic vein. In the two patients without the IRHV, some degree of congestion, which was characterized by alterations of color and engorgement of the remaining inferior right segments (mainly segment 6), was observed in the intraoperative period, but without major bleeding on the raw transected surface. Color-Doppler ultrasonography showed that the portal inflow to segments 5 and 6, as well as the venous outflow in the larger veins that drained these segments, was maintained. Therefore, two patients had maintained outflow through a dominant accessory right hepatic vein, while the other two had no prevailing IRHV. In the absence of an IRHV, the venous outflow might occur through collaterals from the right to left liver as observed in intraoperative color-Doppler and postoperative radiological exams (Figure 1(b)). We strongly recommend intraoperative preresection “venous clamping test” by clamping distal right and middle hepatic veins to evaluate the portal inflow and communicating vessels outflow to future remnant liver segments with color-Doppler ultrasonography. The absence of macroscopic liver congestion or engorgement of the inferior right segments with maintained portal flow may represent compensated venous drainage. Moreover, the absence of bleeding from the raw surface reinforces the compensatory drainage route. When performing resection of the upper segments of the liver with ligation of the middle and right hepatic veins, the surgical team must be prepared to perform vascular reconstruction if major congestion of the right inferior segment occurs leading to transected area excessive bleeding [30, 31]. A main question is whether the increased surgical risk for maximum preservation of liver parenchyma is worthwhile. Resection of the upper segments of the liver implies larger raw surface, requiring longer operative time and higher risk of bleeding. In this article, we report the cases of four patients submitted to upper transversal hepatectomy with neither mortality nor morbidity, no postoperative liver failure, and no need for blood transfusion. No complications were observed in neither the early nor late postoperative period, and the parenchyma-sparing technique favored a reresection in one of the patients due to a segment 3 liver recurrence. There is not enough evidence to conclude that the right upper transversal hepatectomy is better than extended right hepatectomy, and comparative studies including a larger number of cases should be designed; however, we can suggest that it is a feasible alternative with potential benefits.

## 5. Conclusions

Right upper transversal hepatectomy with resection of the distal right and middle hepatic veins and without venous reconstruction is a feasible procedure when an IRHV is present. This parenchymal-sparing technique can be employed, even in the absence of an IRHV, when communicating veins are present draining the right inferior segments to the left hepatic vein. In the absence of an IRHV, even when communicating veins are present, some degree of congestion of the right inferior liver segments might occur in the immediate and early postoperative period; regardless of this, it can be a nonclinically relevant condition. Maximum parenchyma preservation may prevent postoperative liver failure and enable repeated resections.

## Figures and Tables

**Table 1:** Patients' clinical data.

Patient	1	2	3	4
Disease	CRLM	CRLM	CRLM	CRLM
Number of metastases	3	3	1	1
Location (liver segments) and size of metastases	8 (3.5 cm) 1 (1.3 cm) 3 (0.9 cm)	7, 8, 4A (3.9 cm) 5 (4.1 cm) 5 (2 cm)	7, 8, 4A (7.2 cm)	7, 8, 4A (6 cm)
Surgery	RUTH + Seg 1WR + Seg3WR	RUTH + Seg5WR	RUTH	RUTH
IRHV	Present	Present	Absent	Absent
Age (years)	59	69	66	57
Sex	F	M	F	F
BW (kg)	55	71	60	65
Height (meters)	1.59	1.75	1.60	1.65
BMI (kg/m^2^)	21.8	23.2	23.4	23.9
Pre-op chemotherapy	FOLFOX	FOLFOX	—	FOLFOX
Pre-op chemotherapy cycles	2 cycles	3 cycles	—	2 cycles
pRBC	0	0	0	0
FFP	0	0	0	0
Operative time (minutes)	330	420	360	300
EBL (ml)	600	100	400	200
ICU (days)	1	2	2	1
Hospital stay (days)	8	6	7	7
M&M (Dindo-Clavien)	1	1	1	1
Post-op Child-Pugh	5	5	5	5
Follow-up (months)	69	12	39	16

RUTH: right upper transversal hepatectomy, WR: wedge resection, IRHV: inferior right hepatic vein, Seg: liver segment, BW: body weight, BMI: body mass index, CRLM: colorectal liver metastasis, Pre-op chemotherapy: preoperative chemotherapy, Pre-op chemotherapy cycles: number of preoperative chemotherapy cycles, pRBC: packed red blood cells, FFP: fresh frozen plasma, EBL: estimated blood loss, ICU: intensive care unit, MM: morbidity and mortality, Post-op Child-Pugh: postoperative Child-Pugh score.

## Data Availability

The clinical and surgical characteristics of the patients used to support the findings of this study are included within the article, and the data is well documented.
